# Overexpression of Bmi1 in Lymphocytes Stimulates Skeletogenesis by Improving the Osteogenic Microenvironment

**DOI:** 10.1038/srep29171

**Published:** 2016-07-04

**Authors:** Xichao Zhou, Xiuliang Dai, Xuan Wu, Ji Ji, Andrew Karaplis, David Goltzman, Xiangjiao Yang, Dengshun Miao

**Affiliations:** 1 Department of Orthopedics, The First Affiliated Hospital of Soochow University, Suzhou, China; 2 The State Key Laboratory of Reproductive Medicine, the Research Center for Bone and Stem Cells, Department of Anatomy, Histology and Embryology, Nanjing Medical University, Nanjing, China; 3 Department of Fundamentals of Nursing, School of Nursing, Nanjing Medical University, Nanjing, China; 4 The Department of Medicine, McGill University, Montreal, Canada; 5 Rosalind & Morris Goodman Cancer Research Center, Department of Biochemistry, McGill University, Montreal, Quebec, Canada

## Abstract

To investigate whether overexpression of Bmi1 in lymphocytes can stimulate skeletogenesis by improving the osteogenic microenvironment, we examined the skeletal phenotype of EμBmi1 transgenic mice with overexpression of Bmi1 in lymphocytes. The size of the skeleton, trabecular bone volume and osteoblast number, indices of proliferation and differentiation of bone marrow mesenchymal stem cells (BM-MSCs) were increased significantly, ROS levels were reduced and antioxidative capacity was enhanced in EμBmi1 mice compared to WT mice. In PTHrP1–84 knockin (*Pthrp*^KI/KI^) mice, the expression levels of Bmi1 are reduced and potentially can mediate the premature osteoporosis observed. We therefore generated a *Pthrp*^KI/KI^ mice overexpressing Bmi1 in lymphocytes and compared them with *Pthrp*^KI/KI^ and WT littermates. Overexpression of Bmi1 in *Pthrp*^KI/KI^ mice resulted in a longer lifespan, increased body weight and improvement in skeletal growth and parameters of osteoblastic bone formation with reduced ROS levels and DNA damage response parameters. Our results demonstrate that overexpression of Bmi1 in lymphocytes can stimulate osteogenesis *in vivo* and partially rescue defects in skeletal growth and osteogenesis in *Pthrp*^KI/KI^ mice. These studies therefore indicate that overexpression of Bmi1 in lymphocytes can stimulate skeletogenesis by inhibiting oxidative stress and improving the osteogenic microenvironment.

Numerous studies have demonstrated that the endosteal bone marrow microenvironment and cells of the osteoblast lineage play profound roles, in promoting normal hematopoietic stem and progenitor cell function and in malignancy^1^, and a number of cellular and molecular components of the hematopoietic stem cell microenvironment or niche have been identified^1^. In addition, a reciprocal interaction of osteogenic cells and hematopoietic cells has been proposed, in which hematopoietic stem cells (HSCs) also regulate bone marrow mesenchymal stem cell (BM-MSC) induction into osteoblasts thereby participating in the formation of the stem cell niche^2^. Additionally, BM-MSC-like cells regulate B and T cell lymphopoiesis and likely also myelopoiesis^3^. It remains unclear however whether hematopoietic cells regulate skeletogenesis *in vivo* by improving the osteogenic microenvironment.

Bmi1 is a member of the PcG (Polycomb group) family of epigenetic regulators^4^. The Bmi1 locus was initially identified in two independent retroviral tagging screens for oncogenic loci collaborating with the c-Myc proto-oncogene in transgenic mice^5^,^6^. Ablation of the Bmi1 gene in mice leads to posterior transformation, neurological abnormalities, severe hematopoietic defects^7^ and premature osteoporosis^8^. At the cellular level, Bmi1 plays a key role in various cancer stem cells and is required for self-renewal and/or expansion of normal adult hematopoietic, neural, lung, mammary epithelial, and perhaps intestinal stem cells and bone marrow mesenchymal stem cells^8^,^9^,^10^,^11^,^12^,^13^. From a molecular perspective, Bmi1 is an essential subunit of the PRC1 ubiquitin ligase important for silencing expression of Hox genes^14^ and negative cell-cycle regulators such as p16^Ink4a^ and p19^Arf ^15,16. These two cell-cycle regulators are encoded by overlapping transcripts from the gene located on chromosome 9p21, a region frequently deleted in human cancers. p16^Ink4a^ inhibits interaction of cyclin D with cell cycle-dependent kinases 4/6 (CDK4/6) to maintain the Rb tumor suppressor in a hypophosphorylated state. This in turn promotes Rb interaction with E2F and represses transcription of genes required for G1-S transition, leading to cell-cycle arrest at G1. On the other hand, p19^Arf^ acts through the p53 tumor suppressor and regulates expression of genes involved in cell cycle progression, apoptosis and senescence. Bmi1 inhibits expression of both p16^Ink4a^ and p19^Arf^ to regulate tumor-suppressing functions of Rb and p53, respectively. Bmi1 also plays an important role in the regulation of oxidative stress, by reducing the levels of DNA double-strand breaks induced by oxidative stress and promoting DNA repair^17^,^18^. Transgenic mice with specific-overexpression of Bmi1 in lymphocytes, neurons or glial cells displayed susceptibility to development of B cell and T-cell lymphomas, intermediate and anterior lobe pituitary tumors or medulloblastomas^19^,^20^. Previous studies have demonstrated that overexpression of Bmi1 in lymphocytes results in anterior transformation of the axial skeleton along the entire antero-posterior (A-P) axis^21^, but with no change in the number of vertebrae. This phenotype is directly opposite to the posterior transformation which is found along the entire A-P axis of the axial skeleton in Bmi1 null mice^7^. Both phenotypes however exhibit variable penetrance, and it is unknown whether overexpression of Bmi1 in lymphocytes can stimulate appendicular bone development by improving the osteogenic microenvironment.

Parathyroid hormone related peptide (PTHrP) was initially identified as a humoral factor in hypercalcemia of malignancy, a para-neoplastic syndrome characterized by hypercalcemia and hypophosphatemia^22^,^23^. Mouse PTHrP contains 139 residues, with its N-terminal 34 residues homologous to parathyroid hormone (PTH). This PTH-like domain binds to a common PTH/PTHrP receptor (or type I PTH receptor) and stimulates bone formation and/or bone resorption, renal calcium reabsorption, renal phosphate excretion and increased renal production of 1,25 dihydroxyvitamin D. The remaining sequence of PTHrP shows no similarity to PTH and residues 87–107 form an authentic nuclear localization signal (NLS)^24^. Thus, in addition to its PTH-like endocrine, paracrine and autocrine actions through the type I PTH receptor, PTHrP is able to localize directly to the nucleus and exert biological activities independent of PTH. PTHrP is widely expressed in various tissues and influences diverse biological functions, including organ and tissue development; cell survival, migration, proliferation and differentiation; and calcium homeostasis^22^,^25^,^26^. The NLS is important for PTHrP to stimulate cell proliferation and inhibit apoptosis in cultured cells^24^,^27^,^28^. The region C-terminal to the NLS is indispensible for at least some of these activities^29^. To investigate the function of the nuclear localization of PTHrP *in vivo*, we generated a truncation mutant of PTHrP and characterized a PTHrP knock-in (*Pthrp*^KI/KI^) mouse model that carries TGA at codon 85 and thus lacks the mid-region and C-terminal region from residues 85 onward, and expresses only PTHrP1–84. This mutant is defective in nuclear localization. The homozygous mice were slight smaller than wild-type littermates at birth but died by 2–3 weeks of age. The mice displayed characteristic premature aging phenotypes, including osteopenia, kyphosis, cachexia, decreased fat deposition, thin skin, and hyperkeratosis of the epidermis. Strikingly, we found a dramatic decrease of Bmi1 expression and nuclear localization in bone tissues and embryonic fibroblasts^30^. Other studies have shown that nuclear localization of Bmi1 is indispensable for its role in cell transformation and proliferation^31^,^32^. In this study, we assessed whether overexpression of Bmi1 in lymphocytes can correct the premature aging phenotype and the osteoporotic phenotype in the appendicular skeleton of *Pthrp*^KI/KI^ mice.

To examine this issue, we first analyzed the skeletal phenotype of EμBmi1 transgenic mice with overexpression of Bmi1 in lymphocytes to determine whether overexpression of Bmi1 in lymphocytes can stimulate skeletal growth by improving the osteogenic microenvironment. We then crossed EμBmi1 transgenic mice with *Pthrp*^KI/KI^ mice to generate a *Pthrp*^KI/KI^ mouse overexpressing Bmi1 and compared them with *Pthrp*^KI/KI^ and wild-type littermates to clarify whether overexpression of Bmi1 in lymphocytes can correct or improve premature aging and osteoporotic phenotypes occurring in *Pthrp*^KI/KI^ mice.

## Results

### Effect of Bmi1 overexpression in lymphocytes on appendicular bone turnover

**Figure 1:**
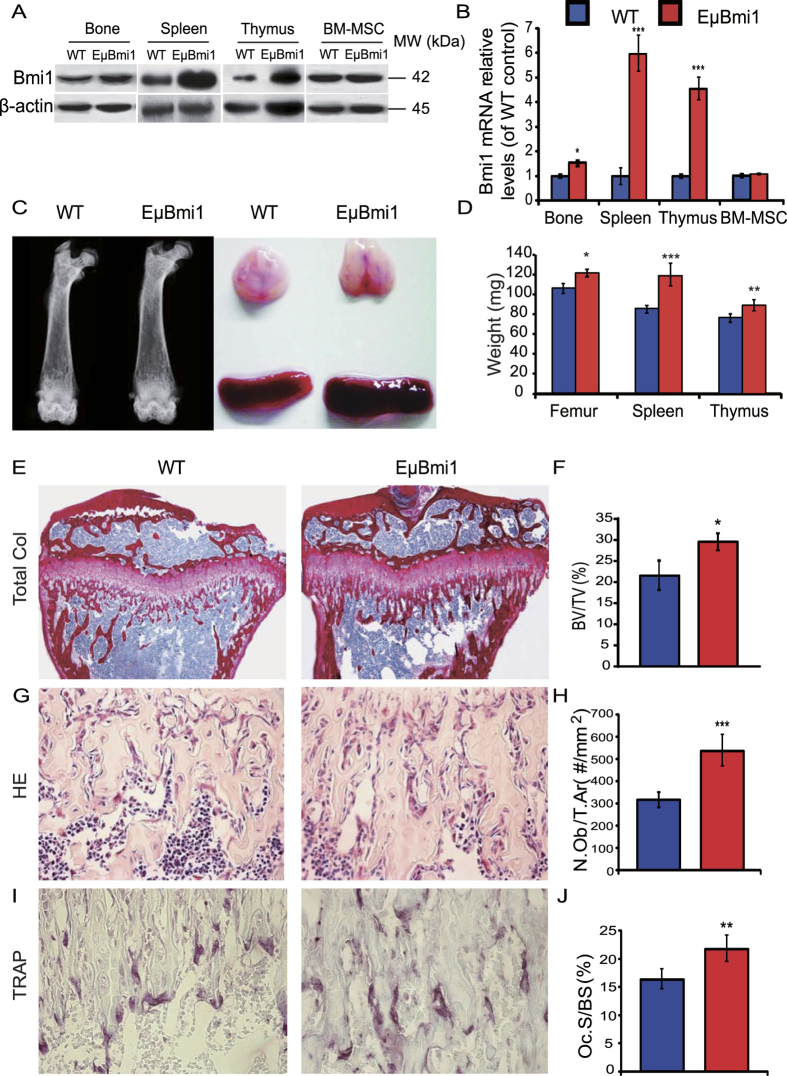
Effect of Bmi1 overexpression in lymphocytes on bone turnover. (**A**) Western blots of bone, spleen and thymus tissue extracts from 8-week-old wild-type (WT) and EμBmi1 transgenic mice for expression of Bmi1. β-actin was used as loading control for Western blots. (**B**) Real-time RT-PCR of bone, spleen and thymus tissue extracts from 8-week-old mice for expression of Bmi1 mRNA. Messenger RNA expression assessed by real-time RT-PCR is calculated as a ratio relative to GAPDH, and expressed relative to WT mice. (**C**) Representative radiographs of the femurs and representative photographs of spleen and thymus from 8-week-old mice. (**D**) Weights of bone, spleen and thymus from 8-week-old mice. Representative micrographs of paraffin-embedded sections of tibiae stained (**E**) histochemically for total collagen, (**G**) with hematoxylin and eosin (**H&E**) and (**I**) histochemically for tartrate-resistant acid phosphatase (TRAP). Magnifications are x50 in E, x400 in G and I. (**F**) Trabecular bone volume (BV/TV, %), (H) Osteoblast number/tissue area (N.Ob/T.Ar, #/mm^2^), and (**J**) Osteoclast surface/bone surface (Oc.S/B.S, %) were determined in 8-week-old mice. Each value is the mean ± SEM of determinations in 8 mice of each genotype. *P < 0.05; **P < 0.01; ***P < 0.001 compared with WT mice.

Previous studies have demonstrated that overexpression of Bmi1 in lymphocytes results in transformation of the axial skeleton, however, it is unknown whether overexpression of Bmi1 in lymphocytes affected bone turnover. At first we confirmed high expression levels of Bmi1 protein and mRNA in bone, spleen and thymus, but not in BM-MSCs in EμBmi1 transgenic mice using Western blots and real-time RT-PCR (Fig. 1A,B). We also found that sizes and weights of appendicular bone, spleen and thymus were increased in 8-week-old EμBmi1 transgenic mice relative to age-matched wild-type mice (Fig. 1C,D). We then examined the skeletal phenotype of EμBmi1 transgenic mice by histology and histochemistry. The results showed that trabecular bone volume, osteoblast number and TRAP positive osteoclast surface in the appendicular skeleton were increased significantly in EμBmi1 transgenic mice compared with their wild-type littermates (Fig. 1E–J). These results indicate that overexpression of Bmi1 in lymphocytes resulted in an augmentation of the appendicular bone volume with accelerated coupled bone turnover, but with increased osteoblastic bone formation exceeding increased osteoclastic bone resorption.

### Effect of Bmi1 overexpression in lymphocytes on the proliferation and differentiation of BM-MSCs

**Figure 2:**
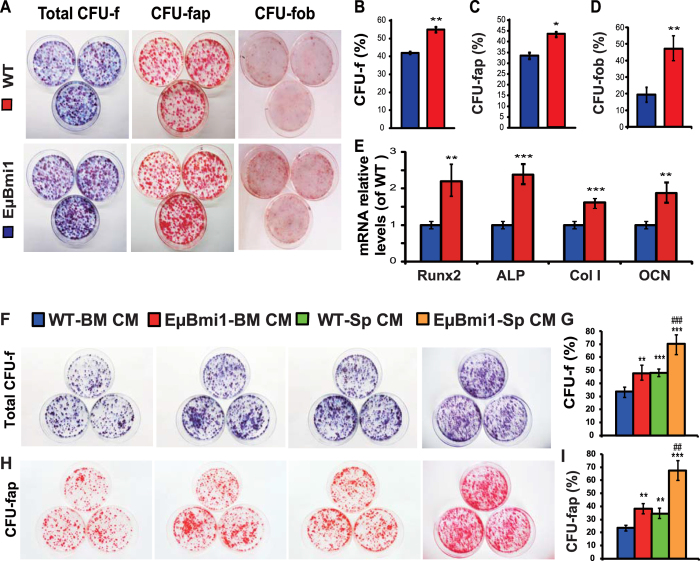
Effect of Bmi1 overexpression in lymphocytes on the proliferation and differentiation of BM-MSCs. (**A**) Primary bone marrow cells from 8-week-old WT and EμBmi1 transgenic mice were cultured *ex vivo* in osteogenic differentiation medium for 14-21 days and resulting cultures were stained with methylene blue for total CFU-f, cytochemically for ALP to show ALP positive CFU-f (CFU-fap), and with alizarin red for calcified nodules (CFU-fob). (**B**) CFU-f areas, (**C**) CFU-fap areas and (**D**) CFU-fob areas relative to culture dish area. (**E**) Real-time RT-PCR of 14-day cultured cell extracts for the expression of Runx2, ALP, type I collagen (Col I) and osteocalcin (OCN). Messenger RNA expression assessed by real-time RT-PCR was calculated as a ratio relative to the GAPDH mRNA level and expressed relative to WT cultures. (**F**) BM-MSCs derived from wild-type mice were cultured with the conditioned media harvested from either bone marrow cells (WT-BM CM/EμBmi1- BM CM) or spleen cells (WT-Sp CM/EμBmi1-Sp CM) cultures derived from WT and EμBmi1 transgenic mice, and resulting cells were stained with methylene blue and cytochemically for ALP to show CFU-f and CFU-fap. (**G**) CFU-f areas, and (**H**) CFU-fap areas relative to culture dish area. *P < 0.05; **P < 0.01; ***P < 0.001 compared with WT cultures.

To determine whether increased osteoblastic bone formation in EμBmi1 transgenic mice was associated with alterations of the proliferation and differentiation of BM-MSCs, we performed CFU-f assays. Cells were stained with methylene blue, cytochemically for ALP, and with alizarin red, to detect total CFU-f, ALP positive CFU-f (CFU-fap) and CFU-f with calcified nodules (CFU-fob), respectively. The results revealed that the percentages of total CFU-f, CFU-fap and CFU-fob areas were increased significantly in EμBmi1 transgenic mice when compared with wild-type mice (Fig. 2A–D). We also examined alterations in expression of genes related to osteogenic cell differentiation. We found that gene expression levels of Runx2, ALP, type I collagen and osteocalcin were up-regulated significantly in osteogenic cells derived from EμBmi1 transgenic mice (Fig. 2E). These results demonstrated that overexpression of Bmi1 in lymphocytes could promote the proliferation and osteogenic differentiation of BM-MSCs.

We then assessed whether the enhanced proliferation and osteogenic differentiation abilities of BM-MSCs from EμBmi1 transgenic mice were associated with osteogenic molecules released by lymphocytes overexpressing Bmi1. For this purpose we harvested the conditioned media from either bone marrow (BM-CM) or spleen cell (Sp-CM) cultures derived from EμBmi1 transgenic mice and wild-type mice, respectively, and added them to cultures of BM-MSCs derived from wild-type mice. We found that the percentages of total CFU-f and CFU-fap areas were increased significantly in the cultures with BM-CM or Sp-CM from EμBmi1 transgenic mice compared with those from wild-type mice (Fig. 2F–J). These results suggest that lymphocytes with Bmi1 overexpression could release osteogenic molecules to stimulate the proliferation and osteogenic differentiation of BM-MSCs.

### Effect of Bmi1 overexpression in lymphocytes on the expression of serum proteins in transgenic mice

**Figure 3:**
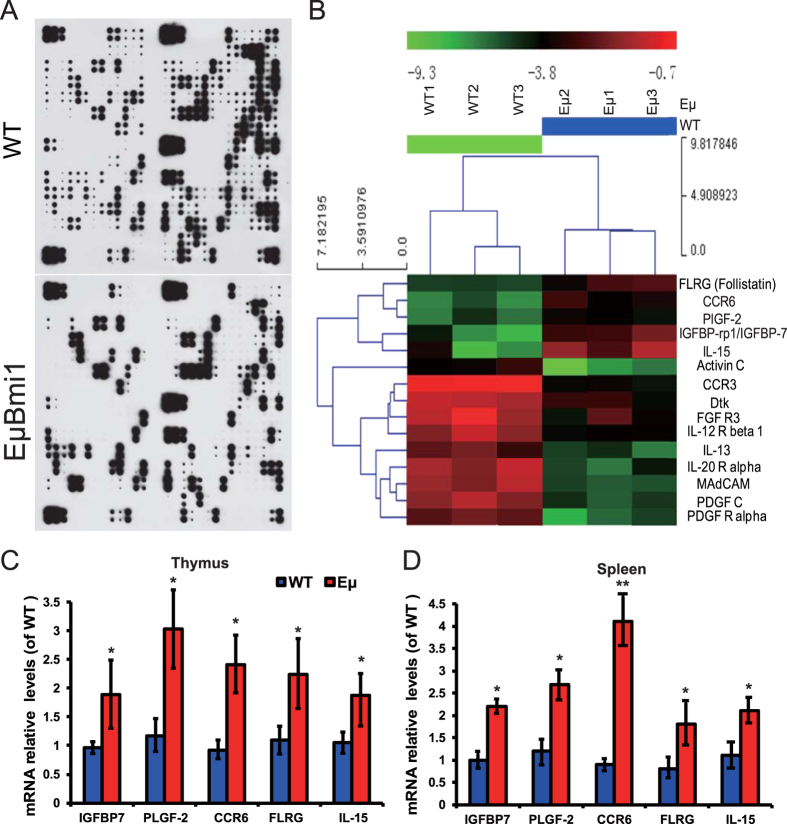
Effect of Bmi1 lymphocyte overexpression on the expression of serum proteins. (**A**) Representative graphs of serum protein arrays of wild-type (WT) and EμBmi1 transgenic mice performed by Raybiotech Biotin Label-based Mouse Antibody Array. (**B**) Graph of cluster analysis. Real-time RT-PCR of (**C**) thymus and (**D**) spleen tissue extracts from 8-week-old WT and EμBmi1 transgenic mice for the expression of follistatin (FLRG), chemokine (C-C motif) receptor 6 (CCR6), placental growth factor 2 (PlGF-2), insulin-like growth factor binding protein 7 (IGFBP-7) and interleukin-15 (IL-15). Messenger RNA expression assessed by real-time RT-PCR was calculated as a ratio relative to the GAPDH mRNA level and expressed relative to WT mice. *P < 0.05; **P < 0.01 compared with WT mice.

To identify the proteins secreted by Bmi1 overexpression lymphocytes that promote osteoblastic bone formation and osteoclastic bone resorption, we conducted protein expression profiling, comparing the differences of serum protein expression levels between the wild-type and EμBmi1 transgenic mice. Cluster analysis was performed and 5 proteins were identified which were significantly increased in the serum of EμBmi1 transgenic mice compared with their wild-type littermates: these were follistatin (FLRG), chemokine (C-C motif) receptor 6 (CCR6), placental growth factor 2 (PlGF-2), insulin-like growth factor binding protein 7 (IGFBP-7) and interleukin-15 (IL-15) (Fig. 3A,B and Table 1). The mRNA expression levels of IGFBP-7, PlGF-2, CCR6, FLRG and IL-15 in thymus and spleen were also significantly up-regulated as demonstrated by real-time RT-PCR (Fig. 3C,D). Whether these factors function as osteogenic factors in the bone microenvironment is currently under investigation. We also detected 11 proteins that were significantly decreased in the serum of EμBmi1 transgenic mice compared with their wild-type littermates (Fig. 3A,B and Table 1).

### Effect of Bmi1 overexpression in lymphocytes on redox homeostasis

**Figure 4:**
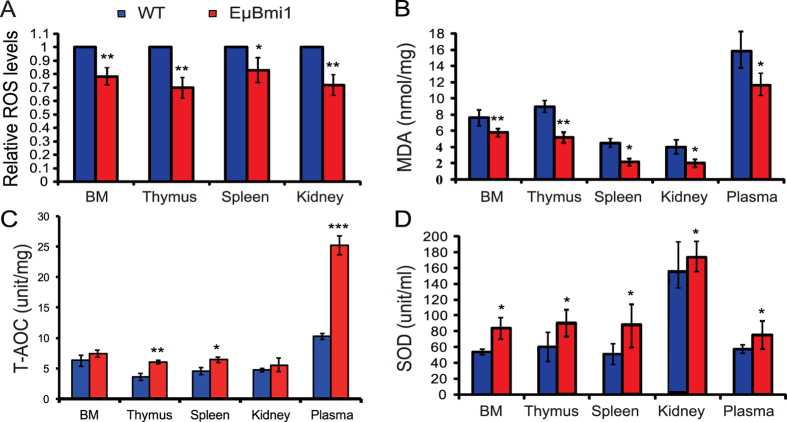
Effect of Bmi1 lymphocyte overexpression on redox homeostasis. (**A**) ROS levels in bone marrow (BM), thymus, spleen and kidney from 8-week-old WT and EμBmi1 transgenic mice. Results of biochemical analysis of BM, thymus, spleen, kidney extracts and plasma from 8-week-old mice for (**B**) malonaldehyde (MDA) content, (**C**) total antioxidant capacity (T-AOC) and (**D**) superoxide dismutase (SOD) activity. Each value is the mean ± SEM of determinations in 5 mice of each genotype. *P < 0.05; **P < 0.01 compared with WT mice.

Previous studies have demonstrated that Bmi1 deficiency led to significant mitochondrial dysfunction accompanied by a sustained increase in ROS. To assess whether the enhanced proliferation and osteogenic differentiation abilities of BM-MSCs caused by Bmi1 overexpression in lymphocytes were associated with reduced ROS production or enhanced antioxidant capacity, we examined ROS levels, malondialdehyde (MDA) content, total antioxidant capacity (T-AOC) and superoxide dismutase (SOD) activity in different tissues from 8-week-old EμBmi1 transgenic mice and their wild-type littermates. We found that ROS levels and MDA content were decreased significantly (Fig. 4A,B), whereas the T-AOC in thymus, spleen, and plasma and SOD activity in bone marrow cells, thymus, spleen, kidney tissues and plasma were increased significantly in EμBmi1 transgenic mice compared with wild-type mice (Fig. 4C,D). These results demonstrated that overexpression of Bmi1 in lymphocytes could maintain a lower levels in ROS in different tissues by enhancing antioxidant capacity.

### Overexpression of Bmi1 in lymphocytes extended the lifespan of *Pthrp* ^*KI/KI*^ mice and improved their growth

**Figure 5:**
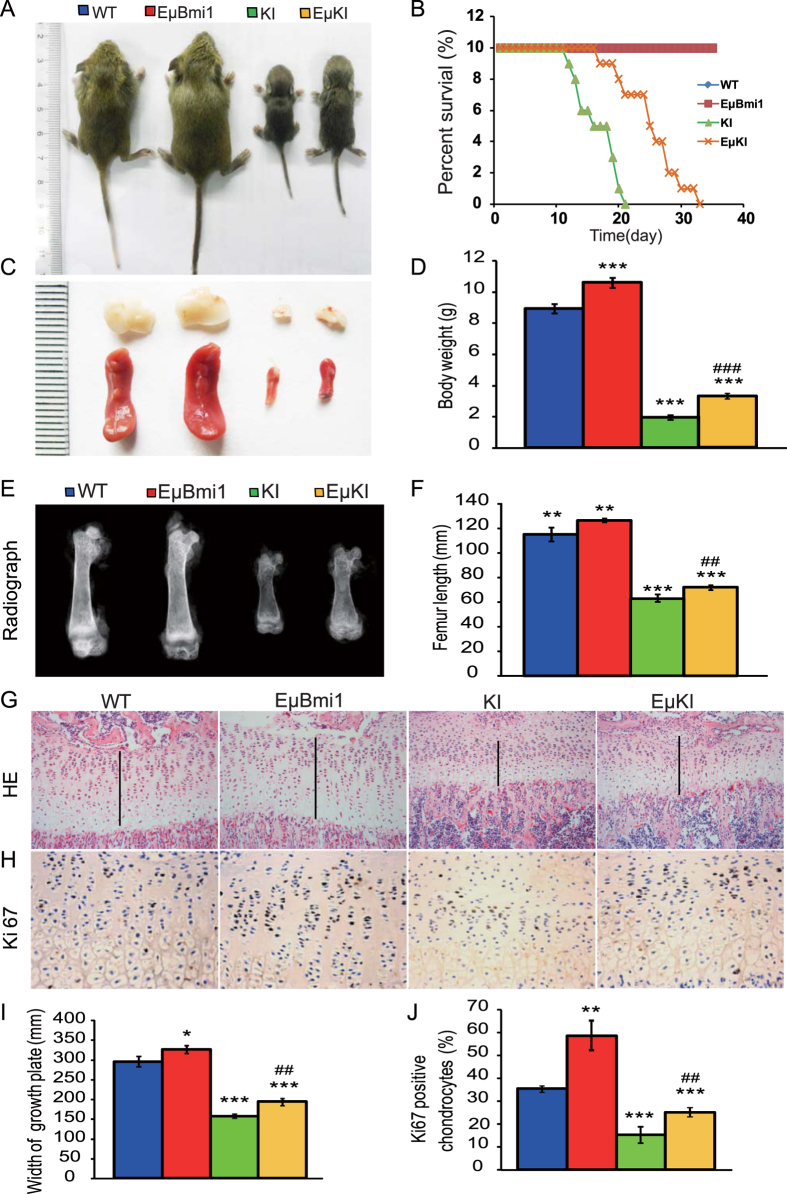
Bmi1 overexpression in lymphocytes extended the lifespan of *Pthrp*^*KI*^/KI mice and improved their growth. (**A**) Whole-body view of 2-week-old WT, EμBmi1, *Pthrp*^KI/KI^ (KI) and EμKI mice. (**B**) Survival curves of 4 genotype mice. (**C**) Representative photographs of spleen and thymus from 2-week-old mice. (**D**) Body weight of 2-week-old mice. (**E**) Representative radiographs of the femurs from 2-week-old mice. (**F**) Femur length of 2-week-old mice. Representative micrographs from paraffin-embedded sections of tibias stained (**G**) with H&E and (**H**) immunohistochemically for Ki67. (**I**) The width of the cartilaginous growth plates. (**J**) The percentage of Ki67-positive chondrocytes relative to total chondrocytes. Magnification is x100 in G and x400 in H. Each value is the mean ± SEM of determinations in 5 mice of each group. *P < 0.05; **P < 0.01; ***P < 0.001 compared with WT mice. ^##^P < 0.01; ^###^P < 0.001 compared with KI mice.

We then compared *Pthrp*^KI/KI^ mice overexpressing Bmi1 in lymphocytes (EμKI) with *Pthrp*^KI/KI^ mice and wild-type littermates. We first examined the effect of Bmi1 overexpression in lymphocytes on the lifespan and skeletal growth of *Pthrp*^KI/KI^ mice. Our results showed that the mean survival time of about 2 weeks of the *Pthrp*^KI/KI^ mice was prolonged to about 3 weeks in the EμKI mice (Fig. 5B). The body size and weight, and sizes of thymus and spleen were significantly increased in EμBmi1 transgenic mice, but were reduced in both *Pthrp*^KI/KI^ and EμKI mice compared to their wild-type littermates; however they were increased significantly in EμKI mice compared to *Pthrp*^KI/KI^ mice (Fig. 5A–D). We also found that the length of long bones, the width of the cartilage growth plates and the percentage of Ki67 positive chondrocytes were clearly increased in EμBmi1 transgenic mice, but were reduced significantly in both *Pthrp*^KI/KI^ and EμKI mice compared to wild-type mice; nevertheless these parameters were increased significantly in EμKI mice compared to *Pthrp*^KI/KI^ mice (Fig. 5E–J). These results demonstrated that overexpression of Bmi1 in lymphocytes can extend the lifespan of *Pthrp*^KI/KI^ mice and improved their growth including skeletal growth.

### Bmi1 overexpression in lymphocytes improved the premature osteoporosis of *Pthrp* ^*KI/KI*^ mice

**Figure 6:**
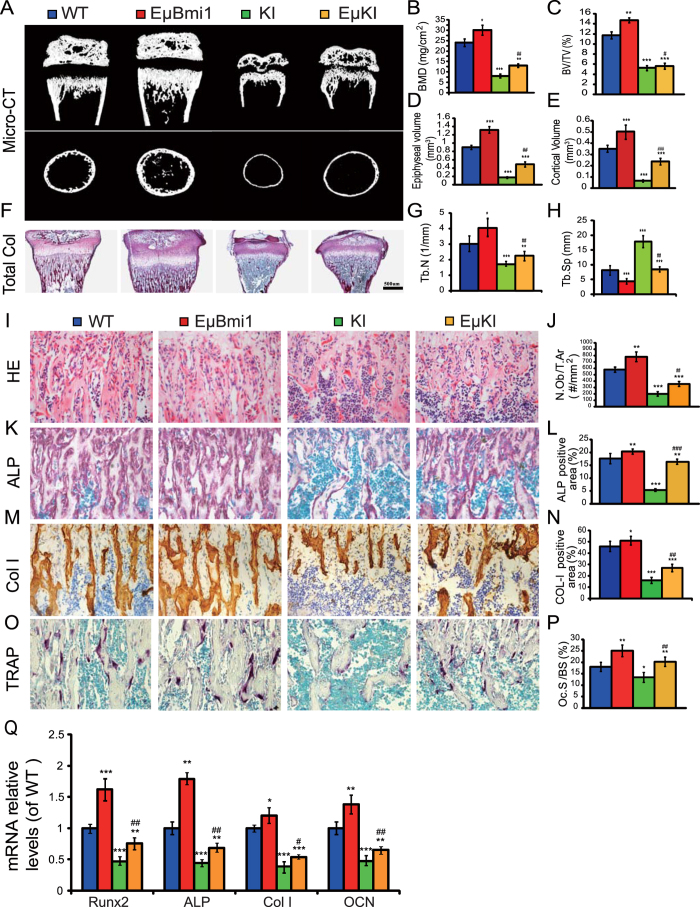
Bmi1 lymphocyte overexpression improved the premature osteoporosis of *Pthrp*^*KI*^/KI mice. (**A**) Representative longitudinal and cross-sectional images of three-dimensional reconstructed distal ends of femurs and midshaft diaphyses from 2-week-old WT, EμBmi1, KI and EμKI mice utilizing micro-CT. Quantitative histomorphometry for (**B**) BV/TV, (**C**) bone mineral density (BMD), (**D**) epiphyseal volume, (**E**) cortical bone volume, (**F**) trabecular number (Tb.N), and (**G**) trabecular separation (Tb.Sp). Representative micrographs of paraffin-embedded sections of tibias stained (**E**) histochemically for total collagen, (**G**) with H&E, and (**K**) histochemically for alkaline phosphatase (ALP) and (**O**) TRAP, and immunohistochemically for Col I (**M**). Magnification is x50 in E, and is x400 in G, K and O. Quantitative histomorphometry for (**J**) N.Ob/T.Ar, the percentage of (**L**) ALP positive and (**N**) Col I positive area, and (**P**) osteoclast surface (Oc.S/B.S) were determined in 2-week-old mice. (**Q**) RT-PCR of 14-day-old bone tissue extracts for the expression of Runx2, ALP, type I collagen (Col I) and osteocalcin (OCN). Messenger RNA expression assessed by RT-PCR was calculated as a ratio relative to the GAPDH mRNA level and expressed relative to WT mice. Each value is the mean ± SEM of determinations in 5 mice of each genotype. *P < 0.05; **P < 0.01; ***P < 0.001 compared with WT mice. ^#^P < 0.05; ^##^P < 0.01: ^###^P < 0.001 compared with KI mice.

To determine whether Bmi1 overexpression in lymphocytes could improve the premature osteoporosis of *Pthrp*^KI/KI^ mice, we examined the effect of Bmi1 overexpression in lymphocytes on bone volume and osteoblastic indices in *Pthrp*^KI/KI^ mice. BMD (Fig. 6A,C), trabecular, epiphyseal and cortical bone volumes (Fig. 6A,C–F), trabecular bone number (Fig. 6A,G), osteoblast numbers (Fig. 6I,J), and ALP (Fig. 6K,L), and type I collagen (Col I) positive areas (Fig. 6M,N) were increased significantly in EμBmi1 transgenic mice, but were reduced significantly in both *Pthrp*^KI/KI^ and EμKI mice compared to wild-type mice; however these parameters were increased significantly in EμKI mice compared to *Pthrp*^KI/KI^ mice. We also examined the alterations in expression of genes related to osteoblastic differentiation. The alterations in the expression of transcripts for Runx2, ALP, type 1 collagen, and osteocalcin, as assessed by real-time RT-PCR, were consistent with the alterations observed by histomorphometry (Fig. 6Q). These results demonstrated that overexpression of Bmi1 in lymphocytes could improve the premature osteoporosis in *Pthrp*^KI/KI^ mice by increasing osteoblastic bone formation.

To determine whether the effect of Bmi1 overexpression in lymphocytes on the alteration of the bone volume of *Pthrp*^KI/KI^ mice is associated with an alteration of osteoclastic bone resorption, we examined the effect of Bmi1 overexpression in lymphocytes on osteoclastic bone resorption of *Pthrp*^KI/KI^ mice by histochemistry and computer-assisted image analysis. We found that the TRAP positive osteoclast surface was increased in EμBmi1 mice, but was decreased significantly in *Pthrp*^KI/KI^ mice compared to wild-type mice. However, it was significantly increased in EμKI mice compared to *Pthrp*^KI/KI^ mice (Fig. 6O,P). These results demonstrated that although Bmi1 overexpression in lymphocytes increased bone volume in *Pthrp*^KI/KI^ mice, this was not by decreasing osteoclastic bone resorption.

### Bmi1 overexpression in lymphocytes reduced the oxidative stress and DNA damage repair responses of *Pthrp* ^*KI/KI*^ mice

**Figure 7:**
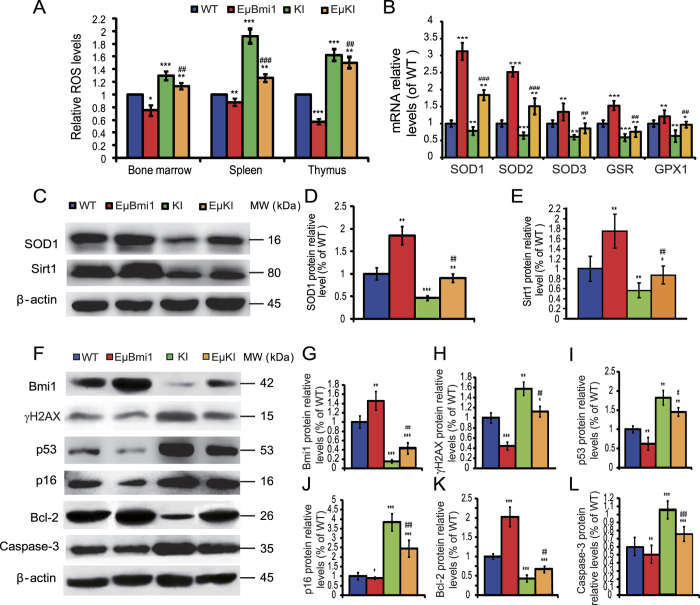
Bmi1 overexpression in lymphocytes reduced the oxidative stress and DNA damage responses of *Pthrp*^*KI/KI*^ mice. (**A**) ROS levels in BM, thymus and spleen from 2-week-old WT, EμBmi1, KI and EμKI mice. (**B**) RT-PCR of bone tissue extracts from 2-week-old mice for expression of SOD1, SOD2, SOD3, glutathione reductase (GSR) and glutathione peroxidase1 (GPX1). Messenger RNA expression assessed by RT-PCR is calculated as a ratio relative to GAPDH, and expressed relative to WT mice. Western blots of long bone extracts from 2-week-old mice for expression of (**C**) SOD1, Sirtuin 1 (Sirt1), (**F**) γ-H2AX, Bmi1, p53, p16, Bcl-2 and caspase-3. β-actin was used as loading control for Western blots. (**D**) SOD1, (**E**) Sirt1, (**G**) Bmi1, (**H**) γ-H2AX, (**I**) p53, (**J**) p16, (**K**) Bcl-2 and (**L**) caspase-3 protein levels were assessed by densitometric analysis calculated as a ratio relative to β-actin protein levels and expressed relative to levels of 2-week-old WT mice. Each value is the mean ± SEM of determinations in 5 mice of each genotype. *P < 0.05; **P < 0.01; ***P < 0.001 compared with WT mice. ^#^P < 0.05; ^##^P < 0.01; ^###^P < 0.001 compared with KI mice.

In order to determine whether the improved *Pthrp*^KI/KI^ premature aging and decreased osteoporosis phenotypes observed after overexpression of Bmi1 in lymphocytes were associated with inhibition of oxidative stress, ROS levels in bone marrow cells, spleen and thymus and the antioxidant enzyme gene and protein expression levels in bone tissue were examined. ROS levels in bone marrow cells, spleen and thymus were reduced in EμBmi1 mice, but were increased in both *Pthrp*^KI/KI^ and EμKI mice compared to wild-type mice, however they were decreased in EμKI mice relative to *Pthrp*^KI/KI^ mice (Fig. 7A). In contrast, the gene expression levels of antioxidant enzymes including SOD1/2/3, GSR and GPX1, and protein expression levels of SOD1 and Sirt1 were up-regulated significantly in EμBmi1 mice, but were down-regulated in *Pthrp*^KI/KI^ mice compared to wild-type mice, however they were up-regulated in EμKI mice relative to *Pthrp*^KI/KI^ mice (Fig. 7B–E). These results demonstrated that PTHrP NLS and C-terminus deficiency induced oxidative stress in various tissues, whereas overexpression of Bmi1 in lymphocytes could reduce oxidative stress in *Pthrp*^KI/KI^ mice.

We then determined whether reducing oxidative stress by overexpression of Bmi1in lymphocytes *in Pthrp*^KI/KI^ mice also inhibited DNA damage responses, by examining expression levels in bone tissue of the DNA double-strand break marker γH2AX, of DNA damage-related proteins Bmi1, p16 and p53, and of apoptotic regulating molecules including Bcl-2 and caspase-3. We found that the expression levels of γH2AX, p16, p53 and caspase-3 were down-regulated in EμBmi1 mice, but were up-regulated in both *Pthrp*^KI/KI^ and EμKI mice compared to wild-type mice; however they were lower in EμKI mice relative to *Pthrp*^KI/KI^ mice (Fig. 7F,H,I, J,L). In contrast, the protein expression levels of Bmi1 and Bcl-2 were up-regulated significantly in bone of EμBmi1 mice, but were down-regulated in bone of *Pthrp*^KI/KI^ and EμKI mice compared to wild-type mice, although they were higher in bone of EμKI mice relative to *Pthrp*^KI/KI^ mice (Fig. 7F,G,K). These results demonstrated that PTHrP NLS and C-terminus deficiency enhanced DNA damage responses in bone tissue whereas overexpression of Bmi1 in lymphocytes could inhibit DNA damage responses in bone of *Pthrp*^KI/KI^ mice.

### Effect of oxidative stress on Bmi1 expression levels and nuclear localization

**Figure 8:**
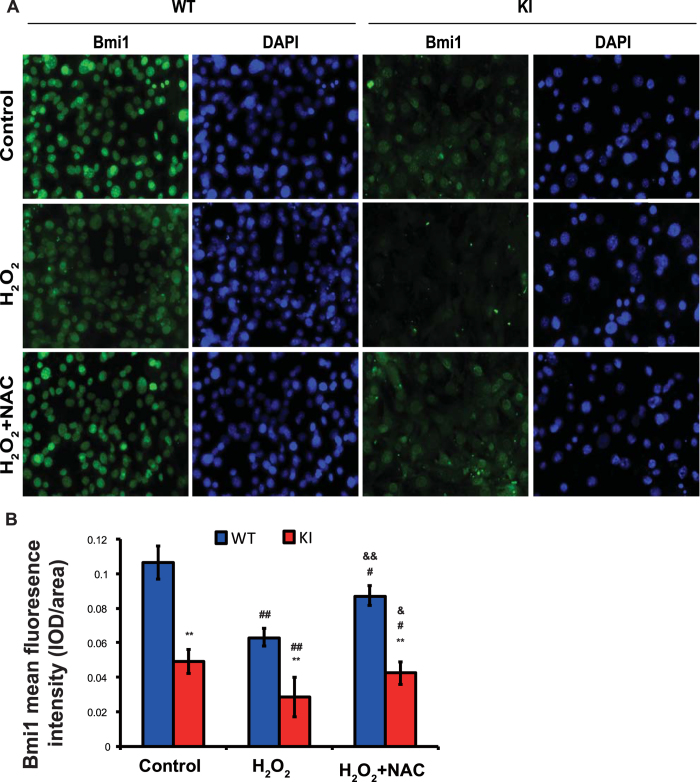
Effect of oxidative stress on Bmi1 expression levels and nuclear localization. (**A**) Representative micrographs of immunofluoresent staining in embryonic fibroblasts from wild-type and *Pthrp*^KI/KI^ mice treated with H_2_O_2_ or with H_2_O_2_ combined with the antioxidant NAC (H_2_O_2_ + NAC). (**B**) Quantified Bmi1 indicates fluoresence intensity (integrated optical density, IOD/ area). Each value is the mean±SEM of determinations in 5 mice of each genotype. **P < 0.01 compared with WT cultures; ^#^P < 0.05; ^##^P < 0.01 compared with control cultures; ^&^P < 0.05; ^&&^P < 0.01 compared with H_2_O_2_ treated cultures.

To assess whether the reductions of Bmi1 expression and nuclear localization observed in *Pthrp*^KI/KI^ mice were associated with oxidative stress, embryonic fibroblasts from wild-type and *Pthrp*^KI/KI^ mice were treated with H_2_O_2_ or H_2_O_2_ combined with the antioxidant NAC, and Bmi1 expression and nuclear localization were examined by immunofluoresent cell staining and computer-assisted image analysis. We found that H_2_O_2_ treatment significantly reduced Bmi1 protein expression in wild-type mice and significantly reduced both Bmi1 protein expression and Bmi1 nuclear localization in *Pthrp*^KI/KI^ mice (Fig. 8A,B). Treatment with NAC rescued H_2_O_2_-induced reductions in Bmi1 expression in wild-type mice and rescued H_2_O_2_-induced reductions of Bmi1 expression and nuclear localization in *Pthrp*^KI/KI^ mice (Fig. 8A,B). These results demonstrated that the decreases in Bmi1 expression and nuclear localization caused by PTHrP NLS and C-terminus deficiency may be mediated via oxidative stress.

## Discussion

BM-MSCs and other cells of the osteoblast lineage appear to form an essential component of the hematopoietic stem cell niche contributing to the proliferation of HSCs and progenitor cells and also regulating B and T cell lymphopoiesis^3^. Conversely HSCs and progenitors appear to regulate BM-MSC induction into osteoblasts. Thus HSCs are able to direct mesenchymal differentiation towards the osteoblastic lineage under basal conditions, but HSCs isolated from animals subjected to an acute stress (e.g., bleeding or 5-FU) were significantly better at inducing osteoblastic differentiation *in vitro* and *in vivo* than were HSCs obtained from control animals. The molecular basis for this activity appeared to be the production of bone morphological protein 2 (BMP2) and BMP6 by HSCs^2^. Additionally the non-adherent mononuclear bone marrow population was able to significantly and robustly provide osteoprogenitors for treatment of osteogenesis imperfecta^33^. We therefore explored in detail whether osteogenesis could be enhanced by hematopoietic cells, notably lymphocytes, that were genetically engineered to convey a potential stimulator of the bone microenvironment, the polycomb protein Bmi1. For this purpose we employed a transgenic mouse model that overexpressed Bmi1 only in lymphocytes, and not in osteogenic cells. Our results revealed that Bmi1 overexpression in lymphocytes enhanced bone turnover and resulted in an augmentation of bone volume with increased osteoblastic bone formation relative to increased osteoclastic bone resorption. We also demonstrated *in vitro* that increased osteogenesis induced by Bmi1 overexpression in lymphocytes resulted from stimulation of the proliferation of BM-MSC and their differentiation into osteoblasts as shown by increased total CFU-f, ALP-positive and calcified CFU-f forming efficiency. Furthermore, osteoblastic differentiation related genes, including Runx2, ALP, type I collagen and osteocalcin, were up-regulated. These results indicate that Bmi1 overexpression in lymphocytes can stimulate osteogenesis.

To examine the mechanism whereby Bmi1 overexpression in lymphocytes stimulates osteogenesis, we showed that the conditioned medium from either bone marrow cell or spleen cell cultures derived from EμBmi1 transgenic mice significantly increased the percentages of total CFU-f and of ALP-positive areas relative to those from wild-type mice. These results suggest that lymphocytes with Bmi1 overexpression can release osteogenic mediators which can stimulate the proliferation and differentiation of BM-MSCs along the osteoblast lineage.

To attempt to identify the osteogenic mediators secreted by lymphocytes overexpressing Bmi1, we performed a serum protein array and cluster analysis and 5 proteins were identified which were significantly increased in EμBmi1 transgenic mice relative to their wild-type littermates. These proteins were identified as follistatin (FLRG), chemokine (C-C motif) receptor 6 (CCR6), placental growth factor 2 (PlGF-2), insulin-like growth factor binding protein 7 (IGFBP-7) and interleukin-15 (IL-15). Previous studies have suggested that 4 of the 5 proteins identified by the protein array, including FLRG, CCR6, PIFG and IGFBP-7, are involved in osteogenesis^34^,^35^,^36^,^37^, whereas IL-15 is a pleiotropic proinflammatory cytokine that appears to help mediate pathological bone loss^38^. The mechanisms whereby these factors function as osteogenic factors in the bone microenvironment is currently under investigation.

Bmi1 null mice display numerous abnormalities including a severe defect in stem cell self-renewal and a shortened lifespan^7^,^8^,^11^,^39^. Besides the de-repression of the Ink4a/Arf locus which mediates many aspects of the Bmi1 deficient phenotypes^40^, protection against oxidative stress emerges as another important pathway downstream of Bmi1. Targeted deletion of Bmi1 resulted in an accumulation of ROS levels, both in knockout mouse models and in human CD34^+^ cells transduced with lentiviral Bmi1 RNAi vectors^18^,^41^. The induction of ROS caused by Bmi1 deficiency could be counteracted by treatment with antioxidants such as N-acetylcysteine (NAC)^18^. Therefore, in view of the fact that Bmi1 functions not only through inhibiting p16 and p19 signalling pathways, but also by protecting against oxidative stress^18^, we asked whether the enhanced proliferation and osteogenic differentiation capabilities of BM-MSCs, caused by Bmi1 overexpression in lymphocytes, were associated with reduced ROS production or enhanced antioxidant capacity. Our results demonstrated that Bmi1 overexpression in lymphocytes significantly reduced ROS levels and MDA content and increased the T-AOC and SOD activities in bone marrow cells, thymus, spleen, and kidney tissues and in plasma. ROS levels may be increased in human mesenchymal stem cells as part of aging, and previous studies have demonstrated that high concentrations of ROS inhibited the proliferation and differentiation of human and mouse mesenchymal stem cells, reduced osteoblast activity and decreased bone formation^42^,^43^,^44^,^45^, however if ROS levels are down-regulated, the proliferative ability of human mesenchymal stem cells is restored^46^. Expression of ROS scavengers is a protective mechanism, and human mesenchymal stem cells express high basal levels of the ROS scavenger glutathione, which enables them to maintain low intracellular ROS levels when exposed to high levels of ROS in culture^47^. Our results suggest that overexpression of Bmi1 in lymphocytes can maintain a lower level in ROS in several tissues including bone tissue by enhancing antioxidant capacity. This may then contribute to enhanced proliferation and differentiation of BM-MSCs, and accelerated osteoblastic bone formation.

In PTHrP1–84 knockin (*Pthrp*^KI/KI^) mice, which are missing the NLS and the C-terminal region of PTHrP, the expression of Bmi1 is reduced, which can potentially mediate the growth arrest and premature osteoporosis observed^30^. We therefore asked whether Bmi1 overexpression in lymphocytes could improve the growth arrest and premature osteoporosis in *Pthrp*^KI/KI^ mice. To address this issue, we crossed EμBmi1 transgenic mice with *Pthrp*^KI/KI^ mice to generate a *Pthrp*^KI/KI^ mice overexpressing Bmi1 in lymphocytes and compared them with *Pthrp*^KI/KI^ and wild-type littermates. We found that overexpression of Bmi1 in *Pthrp*^KI/KI^ mice resulted in mice with a longer lifespan, and with increased body weight and thymus and spleen weight. Chondrocyte proliferation was accelerated resulting in augmented width of growth plates and improvement in skeletal growth. Bone volume was increased due to increased osteoblast number and activity but not due to reduced osteoclastic bone resorption. Our results therefore demonstrated that Bmi1 overexpression in lymphocytes can improve premature aging and skeletal growth retardation, and enhance osteogenesis.

Our previous work has implicated Bmi1 down-regulation and p16 and p21 up-regulation, leading to G1 cell-cycle arrest and senescence^30^ as part of the underlying mechanism for the premature aging and the osteoporotic phenotype caused by PTHrP NLS and C terminus deficiency. In the present study, we asked whether the improvement in premature aging and osteoporosis caused by Bmi1 lymphocyte overexpression in *Pthrp*^KI/KI^ mice was associated with inhibition of oxidative stress. We examined ROS levels in bone marrow cells, spleen and thymus and the gene and protein expression levels of antioxidant enzymes in osseous tissue. ROS levels were found to be increased in bone marrow cells, spleen and thymus and expression levels of antioxidant enzymes including SOD1/2/3, GSR and GPX1 were up-regulated in *Pthrp*^KI/KI^ mice overexpressing Bmi1in lymphocytes. Sirt1, a class III protein deacetylase, is a crucial cell survival protein, which is also involved in combating oxidative stress^48^ and was also up-regulated. We previously demonstrated that Bmi1 deficient mice displayed an osteoporotic phenotype with a significant down-regulation of Sirt1 protein expression, whereas treatment of BM-MSCs with resveratrol, a Sirt1 activator, rescued osteogenesis defects caused by Bmi1 deficiency^8^. Previous work has also shown that Sirt1 haploinsufficiency resulted in a significant reduction in bone mass characterized by decreased bone formation and increased marrow adipogenesis^49^. Recently, we reported that overexpression of Sirt1 in BM-MSCs enhanced osteoblastic bone formation and partially rescued the defects in skeletal growth and osteogenesis in the Bmi1 deficient mice by inhibiting oxidative stress^50^. Our results in the current study therefore are consistent with those observations and indicate that PTHrP NLS and C-terminus deficiency induces oxidative stress in various tissues, whereas overexpression of Bmi1 in lymphocytes reduces oxidative stress in *Pthrp*^KI/KI^ mice at least in part mediated by Sirt1.

We previously reported that in Bmi1 deficient mice, an increase in ROS coincided with an increase in DNA damage and an activation of DNA damage repair pathways; furthermore, treatment with NAC or targeting of CHK2 at least partially restored some of the phenotypes^18^. We therefore asked whether PTHrP NLS and C-terminus deficiency also increased DNA damage and repair responses, as does the down-regulation of Bmi1, and whether the increased DNA damage repair response occurring in *Pthrp*^KI/KI^ mice was reduced by Bmi1 overexpression in lymphocytes. Our results demonstrated that the expression levels of the DNA double-strand break marker γH2AX, and DNA damage-related proteins including p16, p53 and caspase-3 were up-regulated significantly in bone tissue of *Pthrp*^KI/KI^ mice. When Bmi1 expression levels were increased in *Pthrp*^KI/KI^ mice by crossing them with Bmi1 transgenic mice, the DNA damage response pathway-related protein expression levels were down-regulated significantly. These results demonstrated that PTHrP NLS and C-terminus deficiency enhanced DNA damage responses, whereas overexpression of Bmi1 in lymphocytes could inhibit DNA damage responses in *Pthrp*^KI/KI^ mice.

We also assessed whether the reductions of Bmi1 expression and nuclear localization observed in *Pthrp*^KI/KI^ mice was associated with oxidative stress. Thus, embryonic fibroblasts from wild-type and *Pthrp*^KI/KI^ mice were cultured with H_2_O_2_ or H_2_O_2_ combined with the antioxidant NAC, and Bmi1 expression and nuclear localization were examined by immunofluoresent cell staining and computer-assisted image analysis. Our results demonstrated that H_2_O treatment significantly reduced Bmi1 expression levels in wild-type mice and significantly reduced both Bmi1 expression levels and nuclear localization in *Pthrp*^KI/KI^ mice, whereas NAC treatment rescued H_2_O_2_-induced Bmi1 down-regulation in wild-type mice and rescued H_2_O_2_-induced reductions of Bmi1 expression and nuclear localization in *Pthrp*^KI/KI^ mice. Previous studies also demonstrated that H_2_O_2_ can down regulate Bmi1 expression and subsequently up regulate p16 expression in wild-type astrocytes, whereas the treatment of NAC effectively restored Bmi1 expression in ATM- deficient astrocytes^51^. These results suggest that the reductions of Bmi1 expression and nuclear localization caused by PTHrP NLS and C-terminus deficiency may be mediated through oxidative stress. The precise mechanism whereby the PTHrP NLS and C-terminus regulate Bmi1 expression and nuclear localization mediated through oxidative stress remains to be investigated.

In summary, in this study, we employed a genetically modified mouse model that overexpressed Bmi1 only in lymphocytes, but not in osteogenic cells, and demonstrated that Bmi1 overexpressing lymphocytes can release osteogenic factors and reduce ROS levels to stimulate the proliferation of BM-MSCs and their differentiation into osteoblasts, subsequently leading to accelerated osteoblastic bone formation. Furthermore, we generated *Pthrp*^KI/KI^ mice that overexpress Bmi1 in lymphocytes and demonstrated that overexpression of Bmi1 in lymphocytes could stimulate osteogenesis and partially rescue the defects in skeletal growth and osteogenesis in *Pthrp*^KI/KI^ mice. Overexpression of Bmi1 in lymphocytes can therefore improve the osteogenic microenvironment and stimulate osteogenesis at least partly by inhibiting oxidative stress. This may therefore provide a new therapeutic approach for regulating bone formation.

## Materials and Methods

### Derivation and genotyping of mice

EμBmi1 transgenic mice that overexpress Bmi1 in lymphocytes were generated and genotyped as described previously^19^,^21^. The Bmil gene was cloned in a transgene vector linking the gene to the pim-1 promoter, the immunoglobulin enhancer (Eμ) and a Moloney murine leukaemia virus (MoMuLV) Jong terminal repeat (LTR)^21^. The *Pthrp*^KI/KI^ mice (C57BL/6J hybrid background) used in this study were generated and characterized as we previously described^30^. EμBmi1 transgenic mice and *Pthrp*^+/KI^ mice were fertile and were crossed to generate a *Pthrp*^KI/KI^ overexpressing Bmi1 (EμKI) mice. All experimental animals were maintained in a specific pathogen free [SPF] facility and exposed to a 12-h light, 12-h dark cycle. To minimize bias, the animals we used in a group are sex-matched littermates. Animals did not receive any specific treatment. Before sacrifice, animals were anaesthetized with 2% chloral hydrate at a dose of 20 ul/g to minimize suffering. All animal experiments were carried out in compliance with, and approval by, the Institutional Animal Care and Use Committee of Nanjing Medical University. The protocol was approved by the Committee on the Ethics of Animal Experiments of Nanjing Medical University (Permit Number: NJMU2012008).

### Radiography and Micro-computed tomography

For radiography, the left femur from 8-week-old wild-type and EμBmi1 mice or 2-week-old wild-type, EμBmi1, *Pthrp*^KI/KI^ and EμKI mice were removed and dissected free of soft tissue, and fixed overnight in 70% ethanol. X-ray images were obtained using a Faxitron machine (Model 805; Faxitron X-ray Corporation) under constant conditions (22 kV, 4 min exposure), and using Kodak X-Omat TL film (Eastman Kodak Company). For the measurement of bone mineral density (BMD), a PIXImus densitometer (Lunar PIXImus Corporation) was used (5 min image acquisition with a precision of 1% CV for skeletal BMD). The PIXImus software automatically calculated the BMD and recorded data in Microsoft Excel files (Microsoft Corporation). After radiography and BMD measurement, the left femur were analyzed sequentially, as described previously, using micro-computed tomography with a Skyscan 1072 scanner and associated analysis software (Skyscan)^30^. Two-dimensional images were used to generate three-dimensional renderings using the 3D Creator software supplied with the instrument (SkyScan).

### Histology

The right tibias were removed from mice, fixed overnight at 4 °C in PLP fixative (2% paraformaldehyde containing 0.075 M-lysine and 0.01 M-sodium periodate), and histologically processed as described previously^52^. The tibias were decalcified in EDTA and glycerol solution for 7–10 days. The decalcified tibias were dehydrated and embedded in paraffin, after which 5 μm sections were cut on a rotary microtome. The sections were stained with haematoxylin and eosin, or histochemically for total collagen and alkaline phosphatase (ALP) activity and tartrate-resistant acid phosphatase (TRAP), or were used for immunohistochemistry as described below.

### Histochemical staining for collagen, ALP and TRAP

Total collagen was detected in paraffin-embedded sections as described previously^53^. Dewaxed sections were exposed to 1% Sirius red in saturated picric acid for 1 h. After washing with distilled water, sections were counterstained with haematoxylin and mounted with Biomount medium. Enzyme histochemical analysis was performed for the determination of ALP activity, as described previously^54^. Briefly, following pre-incubation overnight with 1% magnesium chloride in 100 mM- Tris-maleate buffer (pH 9.2), dewaxed sections were incubated for 2 h at room temperature in 100 mM-Tris-maleate buffer containing naphthol AS-MX phosphate (0.2 mg/ml; Sigma) dissolved in ethylene glycol monomethyl diethyl ether (Sigma-Aldrich) as a substrate, and fast red TR (0.4 mg/ml; Sigma) as a stain for the reaction product. After washing with distilled water, the sections were counterstained with methyl green nuclear counter stain(Vector Laboratories) and mounted with Kaiser’s glycerol jelly. TRAP enzyme histochemical analysis was performed on paraffin sections using a modification of a previously described protocol^55^. Dewaxed sections were pre-incubated for 20 min in buffer containing 50 mM-sodium acetate and 40 mM-sodium tartrate at pH 5.0. The sections were incubated for 15 min at room temperature in the same buffer containing 2.5 mg/ml of naphthol AS-MX phosphate (Sigma) in dimethylformamide as a substrate, and 0.5 mg/ml of fast garnet GBC(Sigma) as a colour indicator for the reaction product. After washing with distilled water, the sections were counterstained with methyl green and mounted with Kaiser’s glycerol jelly.

### Immunohistochemical staining

Immunohistochemical staining was performed as described previously^52^ with Affinity-purified goat anti-mouse type I collagen antibody (Southern Biotechnology Associates, Birmingham, AL, USA) and polyclonal Rabbit anti-Ki67 (Abcam, Cambridge, UK). Briefly, dewaxed and rehydrated paraffin-embedded sections were incubated with methanol:hydrogen peroxide (1:10) to block endogenous peroxidase activity and then washed in Tris-buffered saline (pH 7.6). The slides were then incubated with the primary antibodies overnight at room temperature. After rinsing with Tris-buffered saline for 15 minutes, tissues were incubated with secondary antibody (biotinylated goat anti-rabbit IgG diluted 1:200, Sigma-Aldrich, St. Louis, MO). Sections were then washed and incubated with the Vectastain Elite ABC reagent (Vector Laboratories, Inc. Ontario, Canada) for 45 minutes. Staining was developed using 3,3-diaminobenzidine (2.5 mg/ml) followed by counterstaining with Mayer’s Hematoxylin.

### Biochemistry assays

Fresh long bones, thymus, spleen, and kidney were removed from mice and homogenated with cold 0.86% physiological saline. After centrifugation at 2500 rpm, 4 °C for 10 min, the supernatants were collected and used for biochemical assays. Total antioxidant activities (T-AOC), malonaldehyde (MDA) and superoxide Dismutase (SOD) content were measured by chemical chromometry according to the manufacturer’s instructions.

### Flow cytometry analysis for intracellular ROS

For analysis of intracellular ROS, total long bone marrow cells, thymus, spleen and kidney cells from 8-week-old wild-type and EμBmi1 mice or 2-week-old wild-type, EμBmi1, *Pthrp*^KI/KI^ and EμKI mice were incubated with 5 mM diacetyldichlorofluorescein (DCFDA) (Invitrogen) and incubated in a shaker at 37 °C for 30 min, followed immediately by flow cytometry analysis in a FACS calibur flow cytometer (Becton Dickinson, Heidelberg, Germany)^18^.

### Serum isolation and protein array analysis

Blood was harvested from 8-week-old wild-type or EμBmi1 transgenic mice and serum was isolated by centrifugation at 3000 rpm for 5 min. Serum protein arrays were performed using Biotin Label-based Mouse Antibody Array I (Raybiotech) according to the manufacturer’s instructions. Briefly, the arrays were blocked, incubated with 100 μL serum samples overnight, then incubated with biotin-conjugated antibodies (1/250) for 2 hours and with HRP-linked secondary antibody (1/1000) overnight. The membranes were incubated with chemiluminescent substrate and exposed to X-ray film for 15 minutes before development. Quantitative array analysis was performed using Array Vision Evaluation 8.0 (GE Healthcare Life Science). Cluster analysis (hierarchical clustering) of the standard value of the pass volcano plot (differential protein expression) was performed and sample proteins of close relationship were clustered together.

### CFU-f assay and cytochemical staining

Tibiae and femurs of 8-week-old wild-type and EμBmi1 mice were removed under aseptic conditions, and bone marrow cells were flushed out with DMEM containing 10% FCS, 50 mg/mL ascorbic acid, 10 mM β-glycerophosphate, and 10^−8^ M dexamethasone. Cells were dispersed by repeated pipetting, and a single-cell suspension was achieved by forcefully expelling the cells through a 22-gauge syringe needle. Total bone marrow cells (10^6^) were cultured in 36-cm^2^ petri dishes in 5 mL of the above-mentioned medium, which was changed every 4 days, and cultures were maintained for 10 to 18 days. At the end of the culture period, cells were washed with PBS, fixed with PLP fixative, and stained with methyl blue or cytochemically for ALP or with alizarin Red for calcified nodules. Following staining, quantitation of total colony (CFU-f) area, of ALP-positive (CFU-Fap) area and alizarin red positive (CFU-fob) area was performed using computer-assisted image analysis as we described previously^52^. CFU-f assays were also performed with 20% conditioned media harvested from either bone marrow cell (BM-CM) or spleen cell (Sp-CM) cultures derived from EμBmi1 transgenic mice and wild-type mice, respectively.

### Western blot analysis

Proteins were extracted from bone tissue, thymus and spleen of 8-week-old wild-type and EμBmi1 mice or from bone tissue from 2-week-old wild-type, EμBmi1, *Pthrp*^KI/KI^ and EμKI mice, and proteins were quantitated using a protein assay kit (Bio-Rad, Mississauga, Ontario, Canada). Protein samples (30 μg) were fractionated by SDS-PAGE and transferred to nitrocellulose membranes. Immunoblotting was carried out as described (9) using primary antibodies against Bmi1 (Millipore), SOD1 (Abcam), Sirt1 (Abcam), γ-H2AX (Ser139) (Cell Signaling Technology), p53 (Cell Signaling Technology), p16 (Santa Cruz Biotechnology, Inc.), Bcl-2 (Santa Cruz Biotechnology,Inc.), caspase-3 (Cell signaling technology), and β-actin (Bioworld Technology, St. Louis Park, MN, USA). For standard Western blotting detection, blots were incubated with HRP-conjugated antibody. Bands were visualized using ECL chemiluminescence (Pierce, Rockford, IL, USA) and quantitated by Scion Image Beta 4.02 (Scion Corporation, NIH).

### Quantitative real-time RT-PCR

RNA was isolated from mouse bone tissue, excluding the growth plate, using Trizol reagent (Invitrogen, Carlsbad, CA, USA) according to the manufacturer’s protocol. Real time RT-PCR were performed as described previously^56^ and the primer sequences used for the real-time PCR are presented in Table 2.

### Computer-assisted image analysis

After H&E staining or histochemical or immunohistochemical staining of sections from each group, images of fields were photographed with a Sony digital camera. Images of micrographs from single sections were digitally recorded using a rectangular template, and recordings were processed and analyzed using Northern Eclipse image analysis software as described^25^,^56^.

### MEF culture and treatment

The primary WT and PTHrP KI MEFs were isolated from E13.5 *Pthrp*^+/KI^ female mice. Briefly, pregnant mice were sacrificed, the uterus exposed, and embryonic mice obtained. The internal tissues and head were removed and then the bodies of the embryos were minced in 1 ml trypsin, and the minced tissue placed in a 37 °C shaker for 1 hour. After 1 h, the digested tissues were plated on 10 cm × 10 cm dishes and cultured in Dulbecco’s modified Eagle’s medium/F-12 medium, supplemented with 10% fetal bovine serum, 5 μg/ml streptomycin and 5 units/ml penicillin. The cells were maintained in a 5% CO_2_ atmosphere at 37 °C. The cells were passaged after 3 days and then every 4 days. F1 offspring cells were digested and placed on chamber slidesfor experimental use. After cell attachment, 200 μM H_2_O_2_ or 200 μM H_2_O_2_ plus 500 μM NAC (N acetyl-semi-ammonia acid) were added to the cultured cells on slide chambers for 2 days. DNA was extracted from the heads of the embryos for genotype identification.

### Immunofluorescent cell staining

The cells on slide chambers were fixed for 40 min, then washed for 10 min in PBS 3 times, blocked for 30 min at room temperature in PBST (10% normal donkey serum, 0.2% Triton X-100) and then washed in PBS for 10 min, 3 times. Bmi1 antibody (Abgent USA) was added into the slide chambers at a dilution of 1 to 50 in PBS (2% donkey serum, 0.2% Triton X-100) and incubated overnight at 4 °C. Slides were then washed in PBS for 10 min, 3 times. Donkey anti-rabbit second antibody (Alexa Fluor F488, life technologies, USA) was then added at a dilution of 1 to 800 in PBS. Slides were then incubated for 1 hour at room temperature, washed in PBS for 10 min, 3 times, and DAPI was then used for nuclear counterstaining. Slides were then mounted and photos taken. The mean fluorescence intensity was analyzed using Image pro plus software which provided the integrated optical density (IOD) over a given area.

### Statistical Analysis

Data from image analysis were presented as mean ± SEM. Statistical comparisons were made using a two-way ANOVA, for two group comparison, and Bonferroni test was used after ANOVA, with P < 0.05 being considered significant.

## Additional Information

**How to cite this article**: Zhou, X. *et al*. Overexpression of Bmi1 in Lymphocytes Stimulates Skeletogenesis by Improving the Osteogenic Microenvironment. *Sci. Rep.* **6**, 29171; doi: 10.1038/srep29171 (2016).

## Figures and Tables

**Table 1:** Serum proteins with significant differences between EμBmi1 and WT mice.

Row	Column	Name	Eμ/WT(fold)	p value
9,10	8	FLRG (Follistatin)	2.574	0.0278
5,6	5	CCR6 (Chemokine receptor 6)	3.762	0.0243
23,24	11	PlGF-2 (Placental growth factor-2)	3.647	0.0266
11,12	24	IGFBP-7 (Insulin-like growth factor-binding protein-7)	2.796	0.0002
17,18	12	IL-15	2.190	0.0008
1,2	11	Activin C	0.117	0.0066
5,6	3	CCR3 (Chemokine receptor 3)	0.099	0.0009
7,8	13	Dtk/Tyro3 (Tyrosine-protein kinase receptor3)	0.121	0.0006
9,10	3	FGF R3 (Fibroblast growth factors receptor3)	0.083	0.0168
17,18	9	IL-12 R beta 1	0.138	0.0031
17,18	10	IL-13	0.278	0.0019
19,20	6	IL-20 R alpha	0.210	0.0367
21,22	7	MAdCAM-1 (mucosal addressin cell adhesion molecule-1)	0.232	0.0313
23,24	6	PDGF C (Platelet-derived growth factor C)	0.099	0.0070
23,24	7	PDGF R alpha (PDGF receptor alpha)	0.059	0.0160

**Table 2:** Sequences of primers employed for RT-PCR.

Name	S/AS	Sequence	Tm (°C)	bp
Runx2	S	GTGACACCGTGTCAGCAAAG	55	356
	AS	GGAGCACAGGAAGTTGGGAC		
ALP	S	CTTGCTGGTGGAAGGAGGCAGG	55	393
	AS	GGAGCACAGGAAGTTGGGAC		
COL-I	S	TCTCCACTCTTCTAGTTCCT	55	269
	AS	TTGGGTCATTTCCACATGC		
OCN	S	CAAGTCCCACACAGCAGCTT	55	370
	AS	AAAGCCGAGCTGCCAGAGTT		
Bmi1	S	ATCCCCACTTAATGTGTGTCCT	55	393
	AS	CTTGCTGGTCTCCAAGTAACG		
SOD1	S	GGTGAACCAGTTGTGTTGTC	56	203
	AS	CCGTCCTTTCCAGCAGTC		
SOD2	S	CAGACCTGCCTTACGACTATGG	56	113
	AS	CTCGGTGGCGTTGAGATTGTT		
SOD3	S	CCTTCTTGTTCTACGGCTTGC	56	142
	AS	TCGCCTATCTTCTCAACCAGG		
GSR	S	GACACCTCTTCCTTCGACTACC	56	116
	AS	CCCAGCTTGTGACTCTCCAC		
GPX1	S	AGTCCACCGTGTATGCCTTCT	56	105
	AS	GAGACGCGACATTCTCAATGA		
FLRG	S	TGCTGCTACTCTGCCAGTTC	62	130
	AS	GTGCTGCAACACTCTTCCTTG		
CCR6	S	CCTGGGCAACATTATGGTGGT	63	123
	AS	CAGAACGGTAGGGTGAGGACA		
PLGF-2	S	GAACGGCTCGTCAGAGGTG	62	187
	AS	ACAGTGCAGATTCTCATCGCC		
IGFBP7	S	CTGGTGCCAAGGTGTTCTTGA	60	90
	AS	CTCCAGAGTGATCCCTTTTTACC		
IL-15	S	ACATCCATCTCGTGCTACTTGT	61	113
	AS	GCCTCTGTTTTAGGGAGACCT		
GAPDH	S	TGGATTTGGACGCATTGGTC	55	211
	AS	TTTGCACTGGTACGTGTTGAT		

Real-time RT-PCR primers used with their name, orientation (S, sense; AS, antisense), sequence, annealing temperature (Tm), and length of amplicon (bp).
